# Depression and Generalized Anxiety as Long-Term Mental Health Consequences of COVID-19 in Iraqi Kurdistan

**DOI:** 10.3390/ijerph20136319

**Published:** 2023-07-07

**Authors:** Perjan Taha, Arazoo Tahir, Fatima Ahmed, Runak Radha, Ari Taha, Shameran Slewa-Younan

**Affiliations:** 1 Psychiatry Unit, Department of Medicine, College of Medicine, University of Duhok, Duhok 42001, Kurdistan Region, Iraq; 2 Community Health, Public Health Department, College of Health and Medical Technology, Shekhan, Duhok Polytechnic University, Duhok 42001, Kurdistan Region, Iraq; arazoo.tahir@dpu.edu.krd; 3 Clinical Biochemistry, Medical Laboratory Technology Department, College of Health and Medical Technology, Shekhan, Duhok Polytechnic University, Duhok 42001, Kurdistan Region, Iraq; fatima.mohammed@dpu.edu.krd; 4 Biology Department, Shorsh General Hospital, Sulaymaniyah Governorate, Sulaymaniyah 46001, Kurdistan Region, Iraq; runakhassani@yahoo.com; 5 Cardiac Center, Erbil, Erbil Governorate, Erbil 44001, Kurdistan Region, Iraq; ariahmedtaha@gmail.com; 6 Translational Health Research Institute, School of Medicine, Western Sydney University, Locked Bag 1797, Penrith, NSW 1797, Australia; s.younan@westernsydney.edu.au

**Keywords:** depression, generalized anxiety, post-COVID-19 symptoms, Iraqi Kurdistan

## Abstract

Coronavirus disease 2019 (COVID-19) has been associated with a variety of psychiatric symptoms. However, COVID-19’s association with psychiatric symptoms after the acute illness phase is not fully understood. Thus, this study sought to examine symptoms of depression and generalized anxiety and associated factors in the period following COVID-19 infection. A cross-sectional study design was conducted in three governorates of the Iraqi Kurdistan region. Face-to-face interviews were held between the period of 15 September and 20 December 2021 with both those who had been infected with COVID-19 and those who had not. Depression symptomology was assessed using the 9-item Patient Health Questionnaire and levels of anxiety were measured using the 7-item Generalized Anxiety Disorder scale. A total of 727 participants were recruited. The bulk of the respondents (*n* = 454) reported having a past COVID-19 infection, of whom a considerable proportion (82%) had mild–moderate symptoms. More than half of the infected respondents (53.3%) stated they were treated at home. The mean score of generalized anxiety was higher among the infected group compared to the non-infected group t(725) = 2.538, *p* = 0.011. Factors such as older age, female gender, unemployment, previous psychological problems, and diabetes mellitus were strongly associated with symptoms of depression and anxiety post-COVID-19 infection. Additionally, anxiety was associated with a longer duration of post-COVID symptoms. The majority of the study population had mild to moderate levels of post-COVID-19 depression and anxiety. Psychological education and interventions are required to reduce the psychological burden of post-COVID-19 symptoms among the general population.

## 1. Introduction

In 2020, the world experienced one of the most serious pandemics in the last two generations. There were daily accounts of the deaths of thousands of people along with news of hundreds of thousands being infected with the highly contagious coronavirus disease (SARS-CoV-2) [1,2]. This new coronavirus (COVID-19) triggered a pandemic, with a higher death and contagiousness ratio than its predecessors [1,2,3]. Due to its rapid spread [4], most governments took swift actions to stop the spread, such as blocking borders, pre-emptive isolation, and quarantine [5]. Despite the success of these policies in reducing the transmission speed, there were significant economic and social consequences [6,7]. Moreover, the impact on health, along with social and economic disruption, was postulated to increase psychological distress and mental illness [8,9]. Research to date has demonstrated mental health concerns related to the COVID-19 pandemic include stress, anxiety and depression, increased alcohol consumption, disordered eating, and uncertainty about the future [10].

Studies conducted in the Iraqi Kurdistan region reported that the pandemic impacted on the mental health and reported fear of the general community, particularly among healthcare workers [11,12,13]. Investigations have demonstrated that previous coronavirus infections such as severe acute respiratory syndrome (SARS) and Middle-East respiratory syndrome (MERS) have been associated with several physical and mental health problems [14,15]. However, the long-term psychological problems of COVID-19 are not well understood [16,17] although there is some evidence to suggest individuals who have recovered from COVID-19 may be at a higher risk of psychological suffering post-infection than others from impacted populations [18]. Moreover, those in occupations were there is a high risk of exposure to infection, such as frontline healthcare workers, along with those who had friends or family members who had been infected, were found to be at a higher risk of psychological distress [19,20].

Given the evidence of elevated rates of reported anxiety, depression, insomnia, and other related social issues, a call for immediate and longer-term mental healthcare provision during the pandemic period was increasingly being noted [21]. Responding to these needs, several countries deployed psychological counselling services [22,23]; however, in developing nations such as Iraq and the Iraqi Kurdistan region, mental health services in the community are absent or at best very limited. Further impacting an accurate response to the communities’ mental health needs are the limited data on common mental health symptoms of anxiety and depression as a result of infection with COVID-19 in the Iraqi Kurdistan region. Consequently, this study aimed to address this dearth of knowledge by examining participants who had been infected with COVID-19 compared to those who had not by looking at the relationship between depression and anxiety with sociodemographic factors and clinical parameters in the post-infection period.

## 2. Materials and Methods

### 2.1. Design and Sampling

A cross-sectional study design was undertaken, with participants from the provinces of Erbil, Duhok, and Sulaymaniyah recruited. The additional province of Halabja was excluded due to limited participant responses and financial restraints precluding ongoing recruitment efforts. A survey was deployed via the Survey Monkey^®^ platform from 15 September 2021 until 20 December 2021 [24].

To ensure the comprehension of the survey by all participants, a team of three interviewers fluent in Kurdish, Arabic, and/or English with data collection experience were engaged and trained in the administration of the measures. Interviewers visited multiple public places, such as restaurants, hospitals, retail malls, and public roads, and approached suitable people to participate. Participants were eligible to participate if they were aged 18 years and older, permanently resided in the Iraqi Kurdistan region, and were able to provide informed consent and participate in a face-to-face interview. A non-discriminative exponential snowball sample distribution technique was used because there are no readily available data to inform a sampling frame and due to it being a cost-effective strategy.

### 2.2. Study Instruments

#### 2.2.1. Sociodemographic and COVID Infection Data

Demographic variables such as age, gender, marital status, employment, smoking, alcohol drinking, history of chronic illnesses (hypertension, diabetes mellitus, respiratory diseases, heart diseases, and renal diseases), and past history of psychiatric disorders were collected. The survey also included some specific questions related to COVID-19 such as vaccination status, number of past infections with COVID-19, severity of COVID-19 symptoms when infected, where they received treatment for their COVID-19 infection, use of vitamins at time of infection, and lastly, the presence of long-term symptoms experienced, their duration, and their functional impact in the post-COVID period.

#### 2.2.2. Patient Health Questionnaire-9

The 9-item Patient Health Questionnaire-9 (PHQ-9) was used to assess depression. It has been proved to be a sensitive and specific tool for screening individuals with suspected depression [25]. Furthermore, the PHQ-9 is the most frequently used tool designed for assessing depression status and grade severity of symptoms in primary care and mental health settings [26,27,28]. Participants were asked to rate their depression experience in the last 2 weeks on a 9-item depression scale ranging from not at all (0 points) to nearly every day (3 points). Clinically, a total PHQ-9 score of 20 and greater suggests severe depression symptoms, 15–19 shows moderately severe depression symptoms, 10–14 indicates moderate depression symptoms, 5–9 indicates mild depression symptoms, and 0–4 indicates having no or minimal depression symptoms, respectively [29]. The PHQ-9 is considered to be reliable for this study sample as the internal consistency is good (α = 0.866).

#### 2.2.3. Generalized Anxiety Disorder-7 Scale

Anxiety was evaluated by the 7-item Generalized Anxiety Disorder (GAD-7) scale [30]. Participants were asked how often they were bothered by the described problem over the last 2 weeks on a four-point scale. Response options were not at all (0 point), several days (1 point), more than half of the days (2 points), and nearly every day (3 points). Scores ranged from 0–21, with those scoring 0–4 as having no or minimal anxiety symptoms, 5–9 as mild anxiety symptoms, 10–14 as moderate anxiety symptoms, and 15 and above as the cut-off for severe anxiety symptoms [30]. The Cronbach’s alpha for GAD-7 in the present study was high (α = 0.897).

### 2.3. Sample Size Calculation

The sample size was estimated using Epi-info and based on a previous study from the Kurdistan region during the COVID-19 pandemic which noted the prevalence of probable depression, anxiety, and stress being 45%, 47%, and 18%, respectively [11]. A sample size of 594 participants was found to be sufficient for achieving a 95% confidence interval for prevalence with ±4% precision. A more conservative, lower precision of 5% was adopted. To account for non-response, bias, and non-representativeness, the sample size was increased to 727.

### 2.4. Data Analyses

Statistical analyses were conducted using SPSS version 23.0 [31]. The demographic data were analyzed using descriptive statistics. Factors were analyzed through binary logistic regression and chi-square test to determine significant values. Means were compared by using independent sample *t* test. *p*-values of <0.05 were considered statistically significant.

### 2.5. Ethical Considerations

Ethics approval for this study was received from the scientific committee of the college of medicine in Duhok University and the local health ethics committee at Duhok General Directorate of Health (reference number 24102021-10-19). Participants were informed about the study objectives, and informed consent was obtained from each one prior to enrollment.

## 3. Results

Table 1 shows the sociodemographic and clinical information of the participants comparing those previously infected with COVID-19 (*n* = 454, 62%) to those not infected (*n* = 273, 38%).

The mean age of the previously infected with COVID-19 participants was 33.48 ± 10.15 years old and their age ranged between 18 and 78 years old and the mean age of the non-infected participants was 33.01 ± 12.38 years old, and the age ranged between 18 and 82 years old. There was no difference in gender between the two groups. Similarly, no difference was noted between the two groups regarding marital status, with 60% of the total sample reporting being married. Around half of the participants were employed (*p* = 0.092). A history of chronic medical illnesses, such as hypertension, diabetes mellitus, respiratory tract diseases, chronic heart diseases, and renal diseases, was also not significantly different between both groups (*p* > 0.05).

However, a reported history of previous psychiatric disorders was found in 43.2% of the COVID-19 infected group compared to only 30.4% in the non-infected group, which was highly statistically significant (*p* = 0.001). Among the participants, 43.2% were still unvaccinated, with the remaining half of participants reporting having received two doses of the COVID-19 vaccine. No statistical difference was noted between the vaccination status and those infected with COVID-19 versus those not (*p* = 0.571).

The clinical parameters of the infected participants with COVID-19 are presented in Table 2.

Around 82.4% of them have a history of COVID-19 infection once and 17.6% twice. Most of the participants (82.4%) had a mild–moderate severity of COVID-19 infection. Around half of the participants self-managed at home, and 39.9% were treated with prescriptions by physicians while still remaining at home. Only 3.3% of the cases were admitted to hospitals. During their COVID-19 infection, 76% of the participants reported using vitamins. About 68.7% of the previously infected persons experienced medical symptoms after recovery from COVID-19 in the post-COVID period. The majority of them (40.7%) suffered from these symptoms for a period of less than 1 month, 21.8% for 1 month or more, and only 11% had these symptoms lasting for 3 months or more.

Finally, over 40% of participants reported a moderate or higher functional impairment due to the COVID-19 infection.

Table 3 shows the probable prevalence rates and symptom severity levels of depression and generalized anxiety among those individuals who reported being infected with COVID-19 compared to those not.

The mean score of depression measured by the PHQ-9 among the previously infected participants (M = 5.84, SD = 5.95) was not statistically different from those not previously infected (M = 5.16, SD = 5.91), t(725) = 1.494, *p* = 0.136. Additionally, the severity levels of depression did not differ significantly between the groups.

The mean score of generalized anxiety of the previously infected group (M = 4.96, SD = 5.27) was significantly higher compared to the non-infected group (M = 3.96, SD = 4.87), t(725) = 2.538, *p* = 0.011. The severity levels of generalized anxiety did not differ significantly across the two groups.

Table 4 presents the sociodemographic and clinical predictors for those scoring moderate to severe levels of depression and generalized anxiety among the COVID-19-infected participants.

Sociodemographic factors predictive of moderate to severe levels of depression were older age (greater than 65 years; OR = 10.795, *p* = 0.033) and female gender (OR = 2.524, *p* = 0.001); however, employment was found to be a protective factor for depression (OR = 0.435, *p* = 0.001). Turning to generalized anxiety, females were twice as likely to score moderate to severe levels of generalized anxiety (OR = 2.398, *p* = 0.003) whereas employment again was a protective factor in reducing the likelihood of this (OR = 0.480, *p* = 0.004).

Among the clinical predictors, having a history of diabetes mellitus increased the risk of clinically significant levels of depression by 27 times (OR = 27.449, *p* = 0.008) and generalized anxiety by 19 times (OR = 19.605, *p* = 0.008). The second strongest predictor for both depression and generalized anxiety was a prior history of psychiatric disorders, with rates increased for depression up to four times (OR = 4.092, *p* < 0.001) and generalized anxiety by three times (OR = 3.534, *p* < 0.001). For generalized anxiety, an additional predictive factor was having a longer duration of post-COVID symptoms of 3 months and more (OR = 2.452, *p* = 0.047). Those who had received both doses of the COVID-19 vaccine had a decreased risk of depression and generalized anxiety of (OR = 0.197, *p* < 0.001) and (OR = 0.283, *p* = 0.001), respectively. Those who received treatment while at home either prescribed by themselves or via instructions from a physician were at a lesser risk of having generalized anxiety (OR = 0.083, *p* = 0.018) and depression (OR = 0.107, *p* = 0.037), respectively.

Table 5 presents acute and ongoing COVID-19 symptoms as predictors for moderate to severe levels of depression and generalized anxiety among the COVID-19-infected participants.

Logistic regression shows that having a history of dyspnea, chest pain, headache, and GIT symptoms were associated with an increased likelihood of clinical depression among the previously infected participants (OR = 1.916, *p* = 0.033), (OR = 1.887, *p* = 0.033), (OR = 2.566, *p* = 0.004), and (OR = 1.950, *p* = 0.017), respectively. However, the only predictor of clinically significant levels of generalized anxiety was having a history of dyspnea (OR = 2.293, *p* = 0.007). Regarding ongoing COVID-19 symptoms, chest pain (OR = 3.173, *p* = 0.024), headache (OR = 2.219, *p* = 0.014), and GIT symptoms (OR = 3.632, *p* = 0.018) were found to be predictive of moderate to severe levels of depression. For those with moderate to severe generalized anxiety levels, ongoing chest pain (OR = 3.506, *p* = 0.014), headache (OR = 2.739, *p* = 0.002), and fatigue (OR = 1.891, *p* = 0.023) were found to be significant predictors.

## 4. Discussion

The present study sought to examine depression and anxiety post-COVID-19 and what factors are associated with clinically significant measures of these mental health conditions in two samples of participants living in North Iraq. To the best of our knowledge, this study represents the first of its kind in comparing samples of participants previously infected with COVID-19 compared to those not at two years post the first wave of infection.

We found no significant associations with sociodemographic characteristics such as age or gender in both the previously and not infected with COVID-19 participants. Interestingly, the majority of the participants (53.3%) infected with COVID-19 reported self-managing their infection or else were treated by physicians but continued to remain at home. Postulated reasons for increased rates of at-home treatment may be related to the misinformation noted on social media [32] warning people about the dangers of being treated in hospital and/or the prohibitive cost of treatment at hospitals, particularly private hospitals.

Our study showed that mild depressive symptoms (26%) were reported by the majority of those previously infected, consistent with the rates found in other countries such as Brazil (26.2%), Italy (31%), and Turkey (18.8%) [33,34,35]. When considering anxiety symptoms, the majority of the previously infected sample had a mild level (22.2%), again similar to previous findings of 22.4% in a prospective cohort study in patients with mild COVID-19 [33]. Compared to the non-infected individuals, those participants previously infected demonstrated higher rates of clinically significant scores for generalized anxiety. This is similar to Guo et al. [36] who also noted COVID-19 patients manifested higher levels of depression and anxiety compared to non-infected controls [36], although interestingly, in our sample, there was a larger portion with more mild depression symptoms. These results seem to support previously reported findings from the earlier SARS outbreak, in which survivors suffered elevated psychological distress lasting 12 months or more following the outbreak [37]. It appears that even among investigations with a shorter period of follow-up (<6 months), COVID-19 infection increased the risk of clinically significant anxiety and depression [17,38]. Wang et al. [39] postulated that a chain mediation model, where the relationship between physical and mental health problems is linked, can help to explain COVID-19 symptoms and mental health outcomes. Furthermore, Wang et al. argued that excessive contradicting health information concerning COVID-19 and its associated physical symptoms may increase the pandemic’s perceived impact, putting people at a higher risk of anxiety, despair, and stress [39].

Our findings regarding the significant sociodemographic predictors of depression and generalized anxiety among the previously infected participants are of particular interest. Specifically, the female gender and unemployment were significant predictors of moderate to severe levels of both depression and generalized anxiety, whereas older age was only predictive of depression. Our findings are in contrast to those of Chang et al. who failed to demonstrate any sociodemographic factors as predictors of depression and anxiety in their study [40]. Our finding that previously infected females were more likely to demonstrate clinically significant symptoms of depression and generalized anxiety is consistent with other investigations of COVID-19 populations [41,42,43,44] and more broadly to higher reported rates of these disorders in females in the general community [45]. There are many postulated reasons to account for such gender differences, ranging from biological differences in brain structures and fluctuations of sex hormones through to psychosocial factors such as women’s increased vulnerability to stressful life events and higher rates of reporting and help-seeking [45]. In addition to these, other factors unique to the COVID-19 circumstances may also play a role in this increased vulnerability of females. For example, the quarantine and stay-at-home mandates potentially increased exposure to domestic violence and conflict in those vulnerable families. Moreover, the central role of women in attending to domestic duties and caring for family would have been associated with greater stress given the highly infectious nature of the pandemic, particularly if coinciding with the women themselves being unwell with COVID-19. Unsurprisingly, unemployed participants were at a higher risk of depression and anxiety, consistent with previous findings [46]. This is particularly notable given, in the case of the Kurdistan region, this finding could be due to political issues and financial austerity [47]. Finally, our finding that older age was associated with clinically significant levels of depression among those previously infected with COVID-19 is supported by past studies including a systematic review, which highlighted that the main risk factor for persisting symptoms was being older [48,49].

When considering chronic physical health conditions, only diabetes mellitus was found to be strongly predictive of mental health outcomes. Notably, we found that having a history of diabetes mellitus increased the risk of depression by 27 times and that of generalized anxiety by 19 times in those previously infected with COVID-19 compared to those not. While it has been well established that patients with diabetes mellitus present with varying degrees of psychological distress and mental health disorders, such as depression and anxiety [50], it seems from our data that COVID-19 seems to further increase this link. While this relationship between chronic health conditions such as diabetes and mental health outcomes in those recovered from a COVID-19 infection has previously been documented [51], there is an increasing recognition of the complex syndemic interaction between other chronic health conditions, pre-existing mental health conditions, and mental health outcomes following infection with COVID-19 [52]. Importantly, we found that a history of previous mental illness was significantly associated with increased depression and anxiety among those who had been infected with COVID-19. This is consistent with other studies that have noted pre-existing psychiatric illness leading to worsening mental health problems during acute COVID-19 infection [53] and post-COVID-19 [35,54]. Almost half of our total sample indicated they were not vaccinated; however, of those who reported having received both doses of the vaccine, we found a significantly lower risk of both anxiety and depression. It is postulated that in such individuals being vaccinated can afford the belief of being protected against severe morbidity and mortality, thus reducing anxiety and improving overall mental health [55]. Indeed, there has been other research to indicate that even with the first dose of vaccination uptake, improvements in mental health outcomes were found among both vaccinated and unvaccinated individuals [56]. Limiting the viral reservoir in the community, becoming less concerned about loved ones, and the increased social and economic activity due to lower illness risk were postulated to account for this [55].

Finally, we sought to examine the impact of both acute and long-term symptoms of COVID-19 on mental health problems. The presence of dyspnea, chest pain, headache, fatigue, and GIT symptoms in both acute or longer stages of the disease significantly led to at least one mental health problem. Similar findings were observed in recent studies which concluded that there was an increased chance of mental health disorders after experiencing COVID-19 physiological symptoms such as fatigue, dyspnea, headache and sleep disturbance, pain, and cough [33,57]. In addition, in a recent meta-analysis, the most common persistent symptoms associated with post-COVID-19 syndromes were fatigue, dyspnea, anosmia, cough, sleep disturbances, arthralgia, headache, and mental health disorders [58].

This study has several strengths. By conducting a first face-to-face interview survey, we were able to ascertain greater accuracy of data and enrolled more participants, especially those with limited literacy. Secondly, by visiting common public places, this modality also attempted to address the issue of selection bias, particularly when past internet-based surveys have been oversubscribed by younger participants. Finally, we utilized well-validated measures of depression and anxiety that have been previously used in the Kurdistan region [59] to identify postulated cases using published cut-off. Nonetheless, both the PHQ-9 and GAD-7 are self-report and not diagnostic interview tools, precluding clinical diagnosis. The cross-sectional nature of this study restricted measures of the population to a specific time point. Finally, the survey was lengthy, which may have discouraged participation.

## 5. Conclusions

In the present study, we found that most of the study participants experienced mild post-COVID-19 symptoms and they managed their infection at home. Additionally, the majority of the participants suffered from mild to moderate depression and anxiety, with anxiety being more prevalent among the infected COVID-19 group. Being female, older age, unemployed, and having past psychiatric problems were all linked to depression and anxiety. Furthermore, those who had symptoms such as dyspnea, headache, chest discomfort, exhaustion, and GIT post-COVID-19 were more likely to suffer from depression and anxiety. We conclude that there is a strong need for ongoing psychological interventions to reduce the psychological burden of post-COVID-19 symptoms among the general population. Efforts from the Ministry of Health, preventative medicine, the universities, media, and engaged community partners are required to ensure the optimal recovery of our population.

## Figures and Tables

**Table 1:** Sociodemographic and clinical data of study population.

Sociodemographic Characteristics	Total (*n* = 727)	Previously Infected (*n* = 454)	Not Infected (*n* = 273)	Comparison		
				t/3	df	*p*
**Age, M (SD)**	33.31 (11.036)	33.48 (10.151)	33.01 (12.383)	0.560	725	0.576
**Gender, *N* (%)**				1.171	1	0.284
Male	366 (53.1)	234 (51.5)	152 (55.7)			
Female	341 (46.9)	220 (48.5)	121 (44.3)			
**Marital status, *N* (%)**				3.359	1	0.072
Unmarried	291 (40)	170 (37.4)	121 (44.3)			
Married	436 (60)	284 (62.6)	152 (55.7)			
**Employment, *N* (%)**				2.943	1	0.092
Employed	376 (51.7)	246 (54.2)	130 (47.6)			
Unemployed	351 (48.3)	208 (45.8)	143 (52.4)			
**Smoking, *N* (%)**				0.385	1	0.559
Yes	138 (19)	83 (18.3)	55 (20.1)			
No	589 (81)	371 (81.7)	218 (79.9)			
**Alcohol drinking, *N* (%)**				0.858	1	0.417
Yes	43 (5.9)	24 (5.3)	19 (7)			
No	684 (94.1)	430 (94.7)	254 (93)			
**Chronic illnesses, *N* (%)**						
**Hypertension**				1.171	1	0.298
Yes	37 (5.1)	20 (4.4)	17 (6.2)			
No	690 (94.9)	434 (95.6)	256 (93.8)			
**Diabetes mellitus, *N* (%)**				5.455	1	0.029
Yes	19 (2.6)	7 (1.5)	12 (4.4)			
No	708 (97.4)	447 (98.5)	261 (95.6)			
**Respiratory diseases, *N* (%)**				0.670	1	0.514
Yes	10 (1.4)	5 (1.1)	5 (1.8)			
No	717 (98.6)	449 (98.9)	268 (98.2)			
**Heart diseases, *N* (%)**				2.269	1	0.149
Yes	12 (1.7)	10 (2.2)	2 (0.7)			
No	715 (98.3)	444 (97.8)	271 (99.3)			
**Renal diseases, *N* (%)**				0.543 ^a^	1	0.717
Yes	8 (1.1)	6 (1.3)	2 (0.7)			
No	719 (98.9)	448 (98.7)	271 (99.3)			
**Previous psychiatric disorders, *N* (%)**				11.754	1	0.001
Yes	279 (38.4)	196 (43.2)	83 (30.4)			
No	448 (61.6)	258 (56.8)	190 (69.6)			
**Received COVID-19 vaccine, *N* (%)**				1.153	2	0.571
Not vaccinated	314 (43.2)	203 (44.7)	111 (40.7)			
One dose	78 (10.7)	47 (10.4)	31 (11.4)			
Two doses	335 (46.1)	204 (44.9)	131 (48)			

M mean, SD Standard Deviation, ^a^ Fisher’s Exact Test, Independent samples *t*-test.

**Table 2:** Clinical parameters of those participants infected with COVID-19 (*n* = 454).

Parameter	Frequency	Percentage %
**Number of past infections with COVID-19**		
Once	374	82.4
Twice	80	17.6
**Severity of COVID-19 symptoms**		
Not sure	23	5.1
Mild–moderate	374	82.4
Severe	46	10.1
Critical	5	1.1
**Management of COVID-19**		
Self-treatment at home	242	53.3
Physician prescription at home	181	39.9
Hospital admission	15	3.3
At outpatient clinics by physician	16	3.5
**Vitamins received during COVID-19**		
Yes	345	76
No	109	24
**Experiencing symptoms in post-COVID period**		
Yes	312	68.7
No	142	31.3
**Duration of post-COVID symptoms after recovery**		
<1 month	185	40.7
1 month	99	21.8
2 months	54	11.9
3 months and more	50	11
**Negative impact of COVID infection on activity engagement**		
Not at all	64	14.1
A little	160	35.2
Moderate level	95	20.9
A lot	98	21.6
A great deal	27	5.9

**Table 3:** Descriptive statistics of depression (measured by PHQ-9) and anxiety (measured by GAD-7) among previously COVID-19-infected versus non-infected participants.

	Infected (*n* = 454) *N* (%)	Non-Infected (*n* = 273) *N* (%)	t/X^2^	df	*p*
**Depression, mean (SD)**	5.84 (5.949)	5.16 (5.908)	1.494	725	0.136
**Severity levels**			3.827	4	0.433
None (0–4)	239 (52.6)	161 (59)			
Mild (5–9)	118 (26)	60 (22)			
Moderate (10–14)	51 (11.2)	30 (11)			
Moderately severe (15–19)	28 (6.2)	11 (4)			
Severe (20–27)	18 (4)	11 (4)			
**Generalized anxiety, mean (SD)**	4.96 (5.269)	3.96 (4.867)	2.538	725	**0.011**
**Severity levels**			4.143	3	0.246
None or minimal (0–4)	267 (58.8)	178 (65.2)			
Mild (5–9)	101 (22.2)	58 (21.2)			
Moderate (10–14)	56 (12.3)	24 (8.8)			
Severe (15+)	30 (6.6)	13 (4.8)			

**Table 4:** Predictors of moderate to severe levels of depression and generalized anxiety among the COVID-19-infected participants (*n* = 454).

	Moderate to Severe Levels of Depression				Moderate to Severe Levels of Generalized Anxiety			
	OR	*p*	95%CI		OR	*p*	95%CI	
			Lower	Upper			Lower	Upper
**Sociodemographic predictors**								
**Age**								
<40 years	1				1			
40–64 years	1.340	0.340	0.735	2.441	1.009	0.978	0.533	1.909
≥65 years	10.795	**0.033**	1.216	95.828	2.440	0.289	0.469	12.711
**Gender**								
Male	1				1			
Female	2.524	**0.001**	1.434	4.444	2.398	**0.003**	1.342	4.286
**Marital status**								
Unmarried	1				1			
Married	0.837	0.497	0.500	1.399	0.866	0.590	0.514	1.461
**Employment**								
Unemployed	1				1			
Employed	0.435	**0.001**	0.268	0.704	0.480	**0.004**	0.292	0.790
**Smoking**								
No	1				1			
Yes	1.899	0.068	0.953	3.785	1.717	0.138	0.840	3.511
**Alcohol drinking**								
No	1				1			
Yes	0.771	0.666	0.237	2.513	0.958	0.943	0.294	3.118
**Clinical predictors**								
**Hypertension**								
No								
Yes	2.923	0.119	0.759	11.257	1.003	0.997	0.222	4.520
**Diabetes mellitus**								
No								
Yes	27.449	**0.008**	2.344	321.415	19.605	**0.007**	2.246	171.134
**Respiratory diseases**								
No								
Yes	1.524	0.778	0.081	28.666	0.333	0.473	0.017	6.700
**Heart diseases**								
No								
Yes	1.319	0.827	0.109	15.937	0.681	0.805	0.032	14.402
**Renal diseases**								
No								
Yes	0.566	0.676	0.039	8.137	0.172	0.305	0.006	4.978
**Previous psychiatric disorders**								
No								
Yes	4.092	**<0.001**	2.150	7.788	3.534	**<0.001**	1.844	6.774
**Received COVID-19 vaccine**								
Not vaccinated						.		
One dose	0.463	0.142	0.166	1.293	0.596	0.334	0.208	1.703
Two doses	0.197	**<0.001**	0.090	0.432	0.283	**0.001**	0.132	0.607
**Number of past infections with COVID-19**	1.209	0.623	0.567	2.579	0.590	0.245	0.242	1.436
**Severity of COVID-19 symptoms**								
Not sure								
Mild–moderate	1.015	0.985	0.228	4.518	2.531	0.296	0.443	14.450
Severe	1.313	0.763	0.223	7.727	3.195	0.261	0.421	24.252
Critical	0.151	0.339	0.003	7.264	0.525	0.710	0.017	15.738
**Management of COVID-19**								
At outpatient clinics								
Self-treatment at home	0.397	0.408	0.044	3.546	0.083	**0.018**	0.011	0.652
Physician prescription at home	0.856	0.892	0.092	7.997	0.107	**0.037**	0.013	0.871
Hospital admission	0.647	0.761	0.039	10.642	0.238	0.307	0.015	3.742
**Vitamins received during COVID-19**								
No								
Yes	0.515	0.091	0.239	1.112	0.538	0.117	0.248	1.169
**Experiencing symptoms in post-COVID period**								
No								
Yes	1.681	0.178	0.789	3.580	2.750	**0.017**	1.199	6.309
**Duration of post-COVID symptoms after recovery**								
<1 month								
1 month	1.166	0.714	0.514	2.647	1.154	0.736	0.503	2.646
2 months	1.058	0.913	0.384	2.913	0.684	0.497	0.228	2.049
3 months and more	2.227	0.076	0.920	5.387	2.452	**0.047**	1.013	5.934

Patient Health Questionnaire-Depression Module total score ≥ 10; GAD, Generalized Anxiety Disorder total score ≥10.

**Table 5:** Acute and ongoing COVID-19 symptoms as predictors for moderate to severe levels of depression and generalized anxiety among the COVID-19-infected participants (*n* = 454).

Symptoms	COVID-19 Symptoms								Post-COVID-19 Symptoms							
	Depression				Generalized Anxiety				Depression				Generalized Anxiety			
	OR	*p*	95%CI		OR	*p*	95%CI		OR	*p*	95%CI		OR	*p*	95%CI	
			Lower	Upper			Lower	Upper			Lower	Upper			Lower	Upper
**Fever**																
No	1				1				1				1			
Yes	0.761	0.422	0.391	1.481	0.933	0.840	0.475	1.832	0.925	0.896	0.287	2.980	0.668	0.507	0.203	2.199
**Cough**																
No	1				1				1				1			
Yes	0.902	0.714	0.519	1.568	0.943	0.837	0.541	1.644	0.929	0.867	0.391	2.206	0.784	0.605	0.313	1.967
**Dyspnea**																
No	1				1				1				1			
Yes	1.916	**0.033**	1.053	3.485	2.293	**0.007**	1.249	4.208	2.179	0.079	0.914	5.195	1.429	0.437	0.581	3.512
**Chest pain**																
No	1				1				1				1			
Yes	1.887	**0.033**	1.052	3.383	1.146	0.669	0.613	2.144	3.173	**0.024**	1.167	8.624	3.506	**0.014**	1.294	9.498
**Headache**																
No	1				1				1				1			
Yes	2.566	**0.004**	1.358	4.848	1.261	0.447	0.693	2.295	2.219	**0.014**	1.173	4.198	2.739	**0.002**	1.442	5.202
**Myalgia**																
No	1				1				1				1			
Yes	0.994	0.984	0.518	1.904	1.619	0.166	0.818	3.203	1.503	0.193	0.814	2.776	1.393	0.302	0.742	2.614
**Fatigue**																
No	1				1				1				1			
Yes	0.747	0.493	0.325	1.718	1.066	0.884	0.448	2.539	1.644	0.068	0.964	2.803	1.891	**0.023**	1.091	3.278
**Sleep disorder**																
No	1				1				1				1			
Yes	1.282	0.398	0.721	2.278	1.183	0.568	0.664	2.107	1.509	0.198	0.806	2.825	1.545	0.178	0.820	2.908
**Anosmia**																
No	1				1				1				1			
Yes	1.054	0.887	0.512	2.168	0.635	0.207	0.314	1.285	1.438	0.399	0.618	3.345	0.728	0.524	0.273	1.935
**Ageusia**																
No	1				1				1				1			
Yes	1.067	0.861	0.516	2.205	1.244	0.547	0.610	2.537	1.132	0.810	0.412	3.110	0.845	0.780	0.259	2.755
**GIT symptoms**																
No	1				1				1				1			
Yes	1.950	**0.017**	1.124	3.383	1.203	0.523	0.682	2.121	3.632	**0.018**	1.251	10.542	2.343	0.121	0.798	6.880

Patient Health Questionnaire-Depression Module total score ≥ 10; GAD, Generalized Anxiety Disorder total score ≥ 10.

## Data Availability

The data that support the findings of this study are available on request from the corresponding author.

## References

[1] Clemente-Suárez V.J., Navarro-Jiménez E., Jimenez M., Hormeño-Holgado A., Martinez-Gonzalez M.B., Benitez-Agudelo J.C., Perez-Palencia N., Laborde-Cárdenas C.C., Tornero-Aguilera J.F. (2021). Impact of COVID-19 pandemic in public mental health: An extensive narrative review. Sustainability.

[2] Wang H., Xia Q., Xiong Z., Li Z., Xiang W., Yuan Y., Liu Y., Li Z. (2020). The psychological distress and coping styles in the early stages of the 2019 coronavirus disease (COVID-19) epidemic in the general mainland Chinese population: A web-based survey. PLoS ONE.

[3] Sohrabi C., Alsafi Z., O’neill N., Khan M., Kerwan A., Al-Jabir A., Iosifidis C., Agha R. (2020). World Health Organization declares global emergency: A review of the 2019 novel coronavirus (COVID-19). Int. J. Surg..

[4] Wu Z., McGoogan J.M. (2020). Characteristics of and important lessons from the coronavirus disease 2019 (COVID-19) outbreak in China: Summary of a report of 72 314 cases from the Chinese Center for Disease Control and Prevention. JAMA.

[5] Devi S. (2020). Travel restrictions hampering COVID-19 response. Lancet.

[6] Linka K., Peirlinck M., Sahli Costabal F., Kuhl E. (2020). Outbreak dynamics of COVID-19 in Europe and the effect of travel restrictions. Comput. Methods Biomech. Biomed. Eng..

[7] Nicola M., Alsafi Z., Sohrabi C., Kerwan A., Al-Jabir A., Iosifidis C., Agha M., Agha R. (2020). The socio-economic implications of the coronavirus pandemic (COVID-19): A review. Int. J. Surg..

[8] Kim H., Hwang Y.H. (2020). Factors contributing to clinical nurse compliance with infection prevention and control practices: A cross-sectional study. Nurs. Health Sci..

[9] Omarini C., Maur M., Luppi G., Narni F., Luppi M., Dominici M., Longo G., Piacentini F. (2020). Cancer treatment during the coronavirus disease 2019 pandemic: Do not postpone, do it!. Eur. J. Cancer.

[10] Altena E., Baglioni C., Espie C.A., Ellis J., Gavriloff D., Holzinger B., Schlarb A., Frase L., Jernelöv S., Riemann D. (2020). Dealing with sleep problems during home confinement due to the COVID-19 outbreak: Practical recommendations from a task force of the European CBT-I Academy. J. Sleep Res..

[11] Othman N. (2020). Depression, anxiety, and stress in the time of COVID-19 pandemic in Kurdistan region, Iraq. Kurd. J. Appl. Res..

[12] Saeed B.A., Shabila N.P., Aziz A.J. (2021). Stress and anxiety among physicians during the COVID-19 outbreak in the Iraqi Kurdistan Region: An online survey. PLoS ONE.

[13] Karim S.K., Taha P.H., Amin N.M.M., Ahmed H.S., Yousif M.K., Hallumy A.M. (2020). COVID-19-related anxiety disorder in Iraq during the pandemic: An online cross-sectional study. Middle East Curr. Psychiatry.

[14] Wang C., Pan R., Wan X., Tan Y., Xu L., McIntyre R.S., Choo F.N., Tran B., Ho R., Sharma V.K. (2020). A longitudinal study on the mental health of general population during the COVID-19 epidemic in China. Brain Behav. Immun..

[15] McGinty E.E., Presskreischer R., Han H., Barry C.L. (2020). Psychological distress and loneliness reported by US adults in 2018 and April 2020. JAMA.

[16] Tahir A.I., Ramadhan D.S., Taha A.A., Abdullah R.Y., Karim S.K., Ahmed A.K., Ahmed S.F. (2021). Public fear of COVID-19 vaccines in Iraqi Kurdistan region: A cross-sectional study. Middle East Curr. Psychiatry.

[17] Shanbehzadeh S., Tavahomi M., Zanjari N., Ebrahimi-Takamjani I., Amiri-Arimi S. (2021). Physical and mental health complications post-COVID-19: Scoping review. J. Psychosom. Res..

[18] Del Rio C., Collins L.F., Malani P. (2020). Long-term health consequences of COVID-19. JAMA.

[19] Dawel A., Shou Y., Smithson M., Cherbuin N., Banfield M., Calear A.L., Farrer L.M., Gray D., Gulliver A., Housen T. (2020). The effect of COVID-19 on mental health and wellbeing in a representative sample of Australian adults. Front. Psychiatry.

[20] Que J., Shi J.D.L., Liu J., Zhang L., Wu S., Gong Y., Huang W., Yuan K., Yan W., Sun Y. (2020). Psychological impact of the COVID-19 pandemic on healthcare workers: A cross-sectional study in China. Gen. Psychiatry.

[21] Petersen E., Wasserman S., Lee S.-S., Go U., Holmes A.H., Al-Abri S., McLellan S., Blumberg L., Tambyah P. (2020). COVID-19–We urgently need to start developing an exit strategy. Int. J. Infect. Dis..

[22] Lai J., Ma S., Wang Y., Cai Z., Hu J., Wei N., Wu J., Du H., Chen T., Li R. (2020). Factors associated with mental health outcomes among health care workers exposed to coronavirus disease 2019. JAMA Netw. Open.

[23] Li W., Yang Y., Liu Z.-H., Zhao Y.-J., Zhang Q., Zhang L., Cheung T., Xiang Y.-T. (2020). Progression of mental health services during the COVID-19 outbreak in China. Int. J. Biol. Sci..

[24] Baker J.D. (2013). Online survey software. Online Instruments, Data Collection, and Electronic Measurements: Organizational Advancements.

[25] Borghero F., Martínez V., Zitko P., Vöhringer P.A., Cavada G., Rojas G. (2018). Screening depressive episodes in adolescents. Validation of the Patient Health Questionnaire-9 (PHQ-9). Rev. Med. Chile.

[26] Kroenke K., Spitzer R.L., Williams J.B. (2001). The PHQ-9: Validity of a brief depression severity measure. J. Gen. Intern. Med..

[27] Levis B., Benedetti A., Thombs B.D. (2019). Accuracy of Patient Health Questionnaire-9 (PHQ-9) for screening to detect major depression: Individual participant data meta-analysis. BMJ.

[28] Spitzer R.L., Kroenke K., Williams J.B., Patient Health Questionnaire Primary Care Study Group (1999). Validation and utility of a self-report version of PRIME-MD: The PHQ primary care study. JAMA.

[29] Manea L., Gilbody S., McMillan D. (2015). A diagnostic meta-analysis of the Patient Health Questionnaire-9 (PHQ-9) algorithm scoring method as a screen for depression. Gen. Hosp. Psychiatry.

[30] Spitzer R.L., Kroenke K., Williams J.B., Löwe B. (2006). A brief measure for assessing generalized anxiety disorder: The GAD-7. Arch. Intern. Med..

[31] SPSS I. (2015). IBM SPSS Statistics for Windows.

[32] Ahmad A.R., Murad H.R. (2020). The impact of social media on panic during the COVID-19 pandemic in Iraqi Kurdistan: Online questionnaire study. J. Med. Internet Res..

[33] Ismael F., Bizario J.C., Battagin T., Zaramella B., Leal F.E., Torales J., Ventriglio A., Marziali M.E., Martins S.S., Castaldelli-Maia J.M. (2021). Post-infection depressive, anxiety and post-traumatic stress symptoms: A prospective cohort study in patients with mild COVID-19. Prog. Neuro-Psychopharmacol. Biol. Psychiatry.

[34] Poyraz B.Ç., Poyraz C.A., Olgun Y., Gürel Ö., Alkan S., Özdemir Y.E., Balkan İ.İ., Karaali R. (2021). Psychiatric morbidity and protracted symptoms after COVID-19. Psychiatry Res..

[35] Mazza M.G., De Lorenzo R., Conte C., Poletti S., Vai B., Bollettini I., Melloni E.M.T., Furlan R., Ciceri F., Rovere-Querini P. (2020). Anxiety and depression in COVID-19 survivors: Role of inflammatory and clinical predictors. Brain Behav. Immun..

[36] Guo Q., Zheng Y., Shi J., Wang J., Li G., Li C., Fromson J.A., Xu Y., Liu X., Xu H. (2020). Immediate psychological distress in quarantined patients with COVID-19 and its association with peripheral inflammation: A mixed-method study. Brain Behav. Immun..

[37] Lee A.M., Wong J.G., McAlonan G.M., Cheung V., Cheung C., Sham P.C., Chu C.-M., Wong P.-C., Tsang K.W., Chua S.E. (2007). Stress and psychological distress among SARS survivors 1 year after the outbreak. Can. J. Psychiatry.

[38] Xie Y., Xu E., Al-Aly Z. (2022). Risks of mental health outcomes in people with covid-19: Cohort study. BMJ.

[39] Wang C., Chudzicka-Czupała A., Tee M.L., Núñez M.I.L., Tripp C., Fardin M.A., Habib H.A., Tran B.X., Adamus K., Anlacan J. (2021). A chain mediation model on COVID-19 symptoms and mental health outcomes in Americans, Asians and Europeans. Sci. Rep..

[40] Chang M.C., Park D. (2020). In Incidence of post-traumatic Stress Disorder after Coronavirus Disease. Healthcare.

[41] Wu Y., Xu X., Chen Z., Duan J., Hashimoto K., Yang L., Liu C., Yang C. (2020). Nervous system involvement after infection with COVID-19 and other coronaviruses. Brain Behav. Immun..

[42] Halpin S.J., McIvor C., Whyatt G., Adams A., Harvey O., McLean L., Walshaw C., Kemp S., Corrado J., Singh R. (2021). Postdischarge symptoms and rehabilitation needs in survivors of COVID-19 infection: A cross-sectional evaluation. J. Med. Virol..

[43] Chen K.-Y., Li T., Gong F.-H., Zhang J.-S., Li X.-K. (2020). Predictors of health-related quality of life and influencing factors for COVID-19 patients, a follow-up at one month. Front. Psychiatry.

[44] Fernández-de-Las-Peñas C., Pellicer-Valero O.J., Navarro-Pardo E., Rodríguez-Jiménez J., Martín-Guerrero J.D., Cigarán-Méndez M. (2021). The number of symptoms at the acute COVID-19 phase is associated with anxiety and depressive long-term post-COVID symptoms: A multicenter study. J. Psychosom. Res..

[45] Farhane-Medina N.Z., Luque B., Tabernero C., Castillo-Mayén R. (2022). Factors associated with gender and sex differences in anxiety prevalence and comorbidity: A systematic review. Sci. Prog..

[46] Shah S.M.A., Mohammad D., Qureshi M.F.H., Abbas M.Z., Aleem S. (2021). Prevalence, psychological responses and associated correlates of depression, anxiety and stress in a global population, during the coronavirus disease (COVID-19) pandemic. Community Ment. Health J..

[47] Ahmad A.B., Cheng Z. (2018). The role of change content, context, process, and leadership in understanding employees’ commitment to change: The case of public organizations in Kurdistan region of Iraq. Public Pers. Manag..

[48] Naharci M.I., Veizi B.G.Y., Katipoglu B., Tasci I. (2021). Psychological burden among community-dwelling older adults with and without a history of a recent Covid-19 infection. Clin. Gerontol..

[49] Nguyen N.N., Hoang V.T., Dao T.L., Dudouet P., Eldin C., Gautret P. (2022). Clinical patterns of somatic symptoms in patients suffering from post-acute long COVID: A systematic review. Eur. J. Clin. Microbiol. Infect. Dis..

[50] Banerjee M., Chakraborty S., Pal R. (2020). Diabetes self-management amid COVID-19 pandemic. Diabetes Metab. Syndr. Clin. Res. Rev..

[51] Demeco A., Marotta N., Barletta M., Pino I., Marinaro C., Petraroli A., Moggio L., Ammendolia A. (2020). Rehabilitation of patients post-COVID-19 infection: A literature review. J. Int. Med. Res..

[52] Saqib K., Qureshi A.S., Butt Z.A. (2023). COVID-19, Mental Health, and Chronic Illnesses: A Syndemic Perspective. Int. J. Environ. Res. Public Health.

[53] Uzunova G., Pallanti S., Hollander E. (2021). Presentation and management of anxiety in individuals with acute symptomatic or asymptomatic COVID-19 infection, and in the post-COVID-19 recovery phase. Int. J. Psychiatry Clin. Pract..

[54] Robillard R., Daros A.R., Phillips J.L., Porteous M., Saad M., Pennestri M.-H., Kendzerska T., Edwards J.D., Solomonova E., Bhatla R. (2021). Emerging New Psychiatric Symptoms and the Worsening of Pre-existing Mental Disorders during the COVID-19 Pandemic: A Canadian Multisite Study: Nouveaux symptômes psychiatriques émergents et détérioration des troubles mentaux préexistants durant la pandémie de la COVID-19: Une étude canadienne multisite. Can. J. Psychiatry.

[55] Perez-Arce F., Angrisani M., Bennett D., Darling J., Kapteyn A., Thomas K. (2021). COVID-19 vaccines and mental distress. PLoS ONE.

[56] Wisnivesky J.P., Govindarajulu U., Bagiella E., Goswami R., Kale M., Campbell K.N., Meliambro K., Chen Z., Aberg J.A., Lin J.J. (2022). Association of Vaccination with the Persistence of Post-COVID Symptoms. J. Gen. Intern. Med..

[57] Sher L. (2021). Post-COVID syndrome and suicide risk. QJM Int. J. Med..

[58] Malik P., Patel K., Pinto C., Jaiswal R., Tirupathi R., Pillai S., Patel U. (2022). Post-acute COVID-19 syndrome (PCS) and health-related quality of life (HRQoL)—A systematic review and meta-analysis. J. Med. Virol..

[59] Taha P.H., Slewa-Younan S. (2020). Measures of depression, generalized anxiety, and posttraumatic stress disorders amongst Yazidi female survivors of ISIS slavery and violence. Int. J. Ment. Health Syst..

